# Myopia progression and associated factors of refractive status in children and adolescents in Tibet and Chongqing during the COVID-19 pandemic

**DOI:** 10.3389/fpubh.2022.993728

**Published:** 2022-10-13

**Authors:** Wujiao Wang, Yongguo Xiang, Lu Zhu, Shijie Zheng, Yan Ji, Bingjing Lv, Liang Xiong, Zhouyu Li, Shenglan Yi, Hongyun Huang, Li Zhang, Fangli Liu, Tong Zhang, Wenjuan Wan, Ke Hu

**Affiliations:** ^1^Ophthalmology Department, The First Affiliated Hospital of Chongqing Medical University, Chongqing, China; ^2^Department of Sports, Health and Arts, Chongqing Municipal Education Commission, Chongqing, China; ^3^Education Department, Physical, Health and Art Education Research Center, National Institute of Education Sciences, Beijing, China; ^4^The First Clinical College, Chongqing Medical University, Chongqing, China

**Keywords:** plateau, outdoor activity time, digital screen time, digital devices, parental awareness, COVID-19

## Abstract

**Objectives:**

To investigate myopia progression and associated factors of refractive status among children and adolescents in Tibet and Chongqing in China during the COVID-19 pandemic.

**Methods:**

A population-based cross-sectional study was conducted to compare rates of myopia and high myopia, axial length (AL), spherical equivalent (SE), outdoor activity time, digital device use, and frequency of visual examinations for children and adolescents affected by myopia in Chongqing and Tibet in 2021.

**Results:**

A total of 2,303 students from Chongqing and 1,687 students from Tibet were examined. The overall prevalence of myopia and high myopia in these two groups were 53.80 and 7.04% vs. 43.86 and 1.30%, respectively in each case. The Chongqing students had a longer AL than the group from Tibet (23.95 vs. 23.40 mm, respectively; *p* < 0.001). The mean SE of the students with myopic parents in Tibet was lower than that of the students in Chongqing with myopic parents (−2.57 ± 2.38 diopters (D) vs. −2.30 ± 2.34 D, respectively) (*p* < 0.001). Conversely, the mean SE of the students from urban areas in Chongqing was lower than that of the students in Tibet (−2.26 ± 2.25 D vs. −1.75 ± 1.96 D, respectively; *p* < 0.001). The Chongqing students exhibited lower SE (−2.44 ± 2.22 D) than their Tibetan counterparts (mean SE: −1.78 ± 1.65 D (*p* = 0.0001) when spending more than 2.5 h outdoors. For example, 61.35% of the students in Tibet spent more than 2.5 h outdoors daily, compared with 43.04% of the students in Chongqing. Correspondingly, the proportion of students using digital devices in Tibet (64.43%) was lower than that in Chongqing (100%). For the latter, 38.62% of the students in Chongqing spent more than 2.5 h online using digital devices compared to 10.49% of the students in Tibet. Greater monitoring of visual status was observed for the Chongqing students (mean SE: −1.90 ± 1.98 D) compared with students in Tibet (mean SE: −2.68 ± 1.85 D) (*p* = 0.0448), with the frequency of optimal examinations being every 6 months. Outdoor activity time was identified as a common risk factor for myopia in both of the populations examined, with odds ratios (ORs) of 1.84 (95% CI: 1.79–1.90) in Chongqing and 0.84 (95% CI: 0.73–0.96) in Tibet. Digital screen time was associated with myopia and high myopia in Chongqing, with ORs of 1.15 (95% CI: 1.08–1.22) and 1.06 (95% CI: 0.94–1.77), respectively. Digital screen time was also found to be a risk factor for high myopia in Tibet (OR: 1.21, 95% CI: 0.77–1.61). The type of digital devices used was also associated with myopia and high myopia in Tibet (OR: 1.33, 95% CI: 1.06–1.68 and OR: 1.49, 95% CI: 0.84–2.58, respectively). Finally, examination frequency was found to correlate with high myopia in the Tibet group (OR: 1.79, 95% CI: 0.66–2.71).

**Conclusion:**

Based on our data, we observed that the prevalence of refractive errors in children and adolescents was significantly lower in Tibet than in Chongqing. These results are potentially due to prolonged outdoor activity time, and the type and time of use for digital devices that characterize the group of children and adolescents from Tibet. It is recommended that parents and children in Chongqing would benefit from increased awareness regarding myopia progression and its prevention.

## Introduction

Myopia has been widely recognized as a major cause of visual impairment. It is predicted by 2050 that nearly half of individuals worldwide will be affected by myopia, with one in five diagnosed with high myopia (1, 2). The countries currently reporting high prevalence of myopia are clustered in East and Southeast Asia. There is a socioeconomic burden associated with cases of myopia due to treatments and monitoring that are needed (3–5). In China, the world's most populous country, the rate of myopia among children and adolescents has continued to rise in recent years. Moreover, the age-adjusted prevalence of myopia in the Chinese population is approximately twice as high as the prevalence rates reported for Caucasian or African populations of children and adolescents (6). By 2050, the prevalence of myopia among children and adolescents aged 3–19 years in China is estimated to be approximately 84% (6). An increased incidence of myopic-related complications is also predicted, and these may involve myopic macular degeneration, retinal detachment, cataracts, and open-angle glaucoma. Furthermore, these conditions may represent important risk factors for irreversible vision loss in cases of high myopia (7).

Prior to the COVID-19 pandemic, at least 2.6 billion people worldwide were experiencing vision impairment. Moreover, a significant proportion of affected individuals were younger than 18 years of age, which is a crucial stage for sensory function growth and intensive eye use (8, 9). Studies have shown that myopia is caused by interactions between both genetic and environmental factors, with identified risk factors, including reduced time outdoors and increased near work (10). During the COVID-19 pandemic, a decrease in outdoor activities and increases in digital screen time due to online courses contributed to the onset and progression of myopia (11, 12). Long-term school closures and home-based study hall may also have impacted the visual status of students. In addition to the effects of visual impairment on reading speed, accuracy, and fluency (13), it can also negatively affect economic development (14). However, if refractive error is corrected, the annual monetary cost for rehabilitation and medical care can be lessened by up to 15% (15).

The Tibetan population in China is distinct from inland populations of China due to its geographical environment, socioeconomic level, and cultural characteristics. Accordingly, congenital heart disease, hypertension, and cataracts have a high incidence and regional characteristics unique to Tibet (16). Some studies have reported that the incidence of myopia among children and adolescents in Tibet is lower than that in the plain areas of China (17–19). However, possible reasons for the observed difference remain unclear. In addition, controlled studies of populations in plain and plateau areas have not been conducted. Qamdo is located in eastern Tibet and has an average altitude of over 3,500 m. It is also characterized by low atmospheric pressure and strong ultraviolet radiation. In contrast, the region of Chongqing is approximately 400 m above sea level and is a much more developed region. Therefore, in this study, we included children and adolescents from two representative regions in China, Qamdo in Tibet and Chongqing, to investigate progression of myopia in plateau vs. plain areas during the COVID-19 pandemic.

## Methods

### Study population

A total of 2,302 students from Chongqing and 1,687 students from Qamdo in Tibet were enrolled in this population-based study. The regions of Chongqing and Tibet represent distinct differences in elevation, at approximately 400 and 3,500 m above sea level, respectively. The rural districts that are encompassed by Tibet include the counties of Chagyab, Markam, and Dengqen. Exclusion criteria for this study were a diagnosis of strabismus or amblyopia. This study included students from first grade of primary school through the senior 2 level. Senior 3 students did not participate due to their preparation for College Entrance Exams. Students from primary schools through junior high schools and their parents/custodians were involved. Each class was randomly selected. If there were less than 25 students per class, students from adjacent classes of the same grade level were enrolled.

### Study design and questionnaire

This population-based cross-sectional study was conducted between January 2021 and May 2021 by adopting stratified cluster sampling. Eligible students and their parents were invited to participate in field tests, questionnaires, and home visits in accordance with the National Student Physique and Health Survey. The questionnaires addressed basic information such as name, age (grade), birth date, gender, heredity, and region, and associated factors such as outdoor activity time, digital device type and time of use, and parental awareness. The Ethics Board of the First Affiliated Hospital of Chongqing Medical University approved this study, and it was conducted in accordance with the tenets of the Declaration of Helsinki. At least one parent or legal guardian of each enrollee was provided with information about the study, and informed consent was signed.

### Visual acuity measurement

The inspection team designated professionals to refine and monitor visual acuity and refractive status for the enrolled students. The equipment used was approved and checked by relevant departments, and received metrological verification and calibration on a regular basis. The logarithmic visual acuity chart used conforms to the national standard (GB11533 standard logarithmic acuity chart). The autorefractometer used meets the requirements of standard criteria (ISO10342 Ophthalmic Instruments-Optometry). Axial length (AL) and mean spherical equivalent (SE) refraction were measured with an optometry unit (Supore, China). SE equals diopter of spherical power (DS) plus 1/2 diopter of cylindrical power (DC). Students exhibiting either of two conditions were judged to be myopic: (a) those wearing orthokeratology lenses or (b) those having a mean uncorrected visual acuity (UCVA) < 5.0 and a mean SE < −0.50D (20). In addition, high myopia was defined as having an SE ≤ −6.00 D (21).

### Statistical analysis

Data analysis was conducted by using GraphPad Prism 8 statistical software (GraphPad Software, Inc., San Diego, CA, USA). Reported mean ± standard deviation values represent data exhibiting normal distribution, whereas median (M) and interquartile range (P25 and P75) values represent data without normal distribution. Binary logistic regression was used to correct for influencing factors. The Kruskal–Wallis and Mann–Whitney tests were adopted to compare variables. Multivariate logistic regression was performed to screen risk factors associated with myopia and high myopia. All statistical tests were two-sided, with *p* < 0.05 considered statistically significant.

## Results

### General information

Overall myopia rates for the adolescent populations examined in Chongqing and Tibet were 53.80 and 43.86%, respectively. The overall high myopia rates were 7.04 and 1.30%, respectively. Compared to Tibet, the myopic rate was higher among each grade of students from Chongqing (Table 1).

**Table 1:** Comparison of myopic rates according to grade for the students examined from Tibet and Chongqing.

**Categories**	**Tibet(N)**	**Chongqing(N)**
P1	20(13.42%)	58(26.61%)
P2	18(11.04%)	60(33.52%)
P3	35(21.88%)	61(34.68%)
P4	43(26.06%)	69(39.66%)
P5	64(36.36%)	92(54.76%)
P6	86(44.33%)	108(63.16%)
J1	87(48.04%)	211(68.73%)
J2	87(50.98%)	242(81.61%)
J3	108(63.53%)	237(77.19%)
H1	164(64.65%)	288(80.83%)
H2	167(74.44%)	294(88.74%)

Myopic rate of each grade in Tibet and Chongqing.

N%, myopia rate; P, primary school; J, junior school; H, high school; “1–6,” grade.

### Gender, heredity, and regional factors

The students in Chongqing had a longer AL than their counterparts in Tibet (23.95 vs. 23.40 mm, respectively; *p* < 0.001). Mean SE with demographic factors is presented in Table 2. Female gender exhibited a greater predisposition for myopia than the male gender, and the mean SE for female in Chongqing (−2.23 ± 1.41 D) was lower than that of the female in Tibet (−1.40 ± 1.88 D) (*p* < 0.001). For students with myopic parents, the mean SE for the Tibet group (−2.57 ± 2.38 D) was lower than that of the Chongqing group (−2.30 ± 2.34 D) (*p* < 0.001). The numbers of students from rural and urban districts in Tibet and Chongqing were 1,176 and 511, and 1,505 and 797, respectively in each case. Accordingly, the mean SE for children and adolescents in the urban areas of Chongqing was lower than for the children and adolescents from Tibet (−2.26 ± 2.25 D and −1.75 ± 1.96 D, respectively; *p* < 0.001 in each case).

**Table 2:** The mean uncorrected visual acuity and spherical equivalent refraction with demographic factors.

	**Tibet**		**Chongqing**		***P***	
	**Categories**	**Mean SE(D) ±SD**	**Categories**	**Mean SE(D) ±SD**	
Gender	Male (*N* = 829)	−1.02 ± 1.56	Male (*N* = 1,142)	−2.19 ± 2.14	< 0.001
	Female (*N* = 858)	−1.40 ± 1.88	Female (*N* = 1,160)	−2.23 ± 1.41	< 0.001
Heredity	With myopic parents (*N* = 548)	−2.57 ± 2.38	With myopic parents (*N* = 789)	−2.30 ± 2.34	< 0.001
	With emmetropic parents (*N* = 1,139)	−2.02 ± 1.98	With emmetropic parents (*N* = 1,499)	−1.95 ± 1.94	< 0.001
Region	Rural (*N* = 1,176)	−0.92 ± 1.30	Rural (*N* = 797)	−1.37 ± 2.09	< 0.001
	Urban (*N* = 511)	−1.75 ± 1.96	Urban (*N* = 1,505)	−2.26 ± 2.25	< 0.001

The mean SE in Chongqing and Tibet were compared by the Mann–Whitney test.

The data obtained for Tibet were based on children and adolescents enrolled from three rural districts. These districts include the counties of Chagyab (*N* = 341, mean SE = −0.94 ± 1.32, *p* < 0.001), Markam (*N* = 401, mean SE = −0.80 ± 1.39, *p* < 0.001), and Dengqen (*N* = 434, mean SE = −0.70 ± 1.40, *p* < 0.001). These regions have minimum altitudes of 3,170, 3,865, and 3,870 m above sea level, respectively. The myopia rates of these three districts were: 24.75, 30.48, and 33.24%, respectively.

### Outdoor activity time

Outdoor activity time was defined as daily exposure to sunlight outdoors (22), with categories of < 2.5 h and ≥2.5 h established for this study. The percentage of Tibetan students who spent more than 2.5 h outdoors each day (61.35%) was higher than that for the Chongqing students (43.04%). Moreover, extended outdoor activity time corresponded with better visual status in both the Chongqing and Tibet populations examined. For example, the students in Tibet and Chongqing who experienced ≥2.5 h outdoors each day had mean SE values of −1.78 ± 1.65 D and −2.44 ± 2.22 D, respectively (*p* = 0.0001).

### Digital devices

The digital devices considered included: televisions, computers, cell phones, and tablet PCs. Digital screen time was defined as the average amount of time students spent on these devices each day (23). The overall proportion of students using digital devices in Tibet (64.43%) was lower than that (100%) in Chongqing. Cell phones were the most commonly used digital devices in both Tibet and Chongqing, with use by 366 (21.70%) and 949 (41.23%) students, respectively. In Tibet, this was followed by televisions (344, 20.39%), tablet PCs (189, 11.20%), and computers (188, 11.14%). In Chongqing, tablet PCs (838, 36.40%), computers (413, 17.94%), and televisions (102, 4.43%) followed. It was further observed that in Chongqing, cell phone users exhibited the lowest SE (−2.53 ± 2.32 D), whereas cell phone users in Tibet exhibited relatively better visual acuity (−1.88 ± 1.87 D) (*p* < 0.001). Computer users demonstrated the best visual acuity in Chongqing and Tibet, with the mean SE of the Chongqing group (−1.46 ± 1.38 D) being higher than that of the Tibet group (−1.52 ± 1.76 D) (*p* < 0.0001).

Classification of utility time on digital devices was consistent with that of outdoor activity time. For example, students in both Chongqing and Tibet exhibited the greatest visual status when they reported use of digital devices for less than 2.5 h/day. In contrast, 38.62% students in Chongqing used digital devices for more than 2.5 h/day, whereas this percentage was 10.49% for the students in Tibet. Similarly, the SEs for these two groups were −2.52 ± 2.09D and −2.48 ± 2.07 D, respectively (*p* = 0.004).

### Parental awareness of myopia prevention

**Figure 1:**
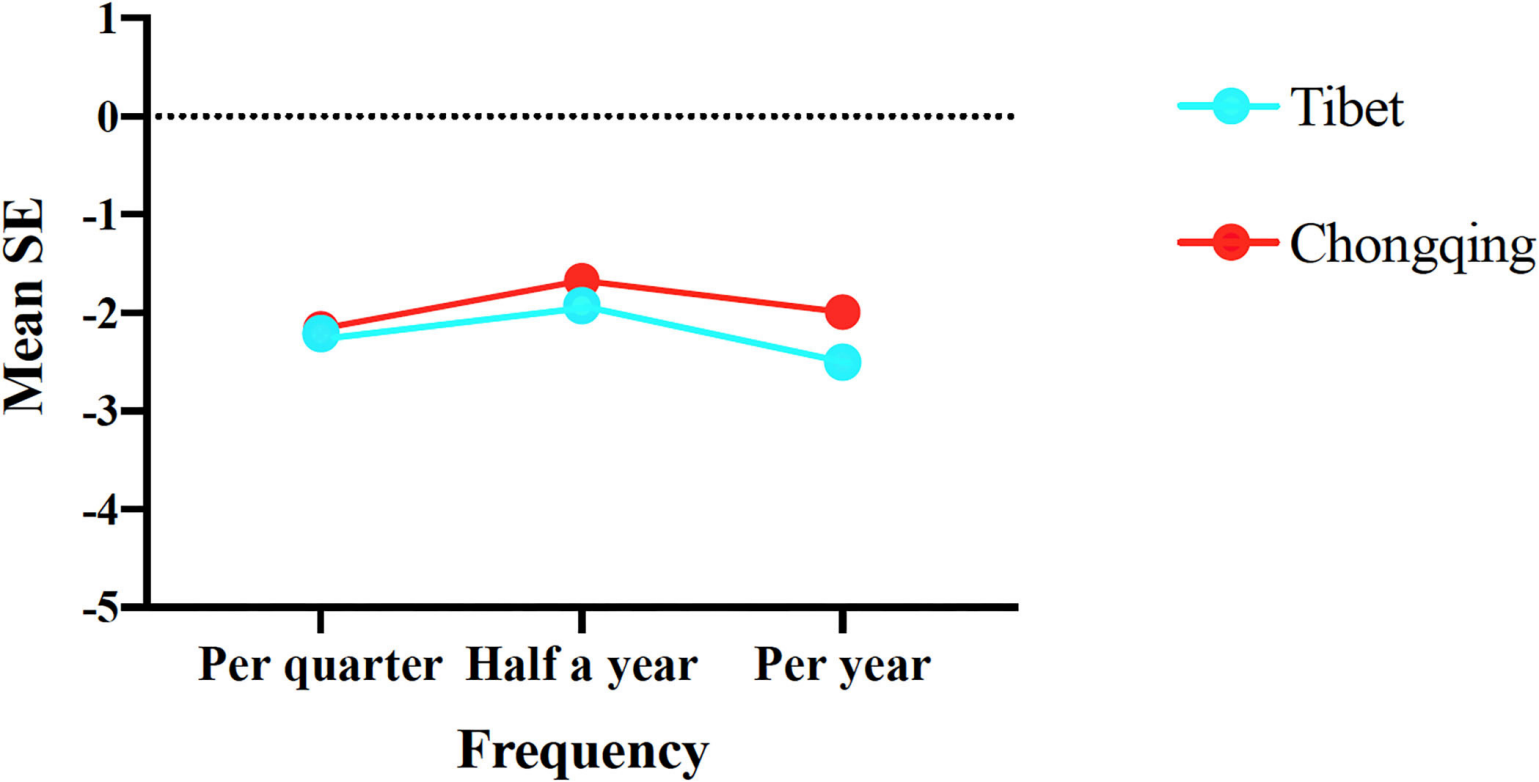
Mean SE according to parental awareness for the student groups from Chongqing and Tibet. The Kruskal–Wallis test was used to compare mean SE of examination frequency in Chongqing and Tibet.

Parental awareness is one of the factors associated with myopia prevention. For our cohort, the frequency of visual examinations ranged from once per quarter, to twice a year, and to once a year. In Chongqing, the proportions of students for these three frequencies were 389 (16.90%), 1,086 (47.18%), and 827 (35.92%), respectively; meanwhile, the frequencies for Tibet were 169 (10.01%), 149 (8.83%), and 155 (9.19%), respectively. The students undergoing a visual examination twice a year exhibited the highest SE (which was the most appropriate recheck period), followed by the quarterly and yearly frequencies (Figure 1). The students in Chongqing who received a visual examination twice a year had a higher SE (−1.90 ± 1.98 D) than the students with the same examination frequency in Tibet (mean SE: −2.68 ± 1.85 D (*p* = 0.0448).

### Multivariate logistic regression analysis

Multivariate logistic regression identified outdoor activity time as a common risk factor for myopia in both of the adolescent populations from Chongqing and Tibet [OR: 1.84 (95% CI: 1.79–1.90) vs. 0.84 (95% CI: 0.73–0.96), respectively] (Tables 3, 4). Digital screen time was associated with myopia and high myopia in Chongqing, with ORs of 1.15 (95% CI: 1.08–1.22) and 1.06 (95% CI: 0.94–1.77), respectively (Table 3). However, digital screen time was only a risk factor for high myopia in Tibet (OR: 1.21, 95% CI: 0.77–1.61), whereas use of digital devices was associated with both myopia and high myopia in Tibet (OR: 1.33, 95% CI: 1.06–1.68; OR: 1.49, 95% CI: 0.84–2.58). Examination frequency was also identified as a risk factor for high myopia in Tibet (OR: 1.79, 95% CI: 0.66–2.71) (Table 4). Meanwhile, in Chongqing, outdoor activity time, digital screen time, digital device use, and examination frequency were identified as risk factors for AL. In Tibet, the only risk factors for AL were digital screen time and examination frequency (Tables 3, 4).

**Table 3:** Multivariate logistic regression results for myopia and high myopia in Chongqing.

**Variable (Chongqing)**	**Myopia (*****N*** = **1,239)**			**High myopia (*****N*** = **162)**			**Axial length(mm)**		
	**Odds ratio (95% CI)**	***P***	**β**	**Odds ratio (95% CI)**	***P***	**β**	**95% CI**	***P***	**β**
Outdoor activity time	1.84 (1.79–1.90)	< 0.0001	0.17	0.84 (0.73–0.96)	< 0.0001	−0.17	−0.14 to 0.06	< 0.0001	0.02
Digital screen time	1.15 (1.08–1.22)	< 0.0001	0.19	1.0 6 (0.94–1.77)	< 0.0001	0.05	0.06 to 0.13	0.0011	0.02
Digital devices	0.85 (0.77–0.88)	< 0.0001	−0.16	0.63 (0.51–0.79)	< 0.0001	−0.44	−0.18 to 0.06	< 0.0001	0.03
Examination frequency	0.82 (0.76–0.88)	< 0.0001	−0.19	0.91 (0.80–1.05)	< 0.0001	−0.09	−0.13- to 0.05	< 0.0001	0.02

CI, confidence interval; OR, odds ratio.

Odds ratio (95% confidence interval) of myopia (SE < 0.50 D) and high myopia (SE ≤ −6.00 D), comparing the outdoor activity, digital screen time, digital devices and examination frequency. OR = 1, no correlation between exposure and outcome; OR > 1, exposure contributes to outcome; OR < 1, exposure prevents the outcome.

**Table 4:** Multivariate logistic regression results for myopia and high myopia in Tibet.

**Variable(Tibet)**	**Myopia (*****N*** = **740)**			**High myopia (*****N*** = **22)**			**Axial length (mm)**		
	**Odds ratio (95% CI)**	***P***	**β**	**Odds ratio (95% CI)**	***P***	**β**	**(95% CI)**	***P***	**β**
Outdoor activity time	1.04 (0.92–1.18)	= 0.0013	0.04	0.77 (0.44–1.23)	= 0.0021	−0.26	−0.16 to 0.01	0.0048	−0.07
Digital screen time	0.93 (0.81–1.09)	= 0.0001	−0.07	1.21 (0.77–1.61)	= 0.0024	0.11	0.05 to 0.20	0.0041	0.08
Digital devices	1.33 (1.06–1.68)	< 0.0001	0.03	1.49 (0.84–2.58)	= 0.0001	0.40	−0.12 to 0.02	0.0089	−0.05
Examination frequency	0.93 (0.65–1.31)	< 0.0004	−0.07	1.79 (0.66–2.71)	=0.0001	0.58	−0.15 to 0.25	0.0044	0.05

CI, confidence interval; OR, odds ratio.

Odds ratio (95% confidence interval) of myopia (SE < 0.50 D) and high myopia (SE ≤ −6.00 D), comparing the outdoor activity, digital screen time, digital devices and examination frequency. OR = 1, no correlation between exposure and outcome; OR > 1, exposure contributes to outcome; OR < 1, exposure prevents the outcome.

## Discussion

In this population-based cross-sectional study of incidence of myopia among children and adolescents in Tibet and Chongqing, the total myopia rate of Tibetan children and adolescents (*N* = 1,687) living in a plateau region was higher than that of Han children and adolescents living in Chongqing (*N* = 2303) during the recent COVID-19 outbreak. The mean SE of children and adolescents in urban areas of Chongqing was also lower than that in Tibet (−2.26 ± 2.25 D vs. −1.75 ± 1.96 D, respectively) (*p* < 0.001). However, it was also observed that the mean SE of students with myopic parents in Tibet (−2.57 ± 2.38 D) was lower than that in Chongqing (−2.30 ± 2.34 D) (*p* < 0.001). Overall, the percentage of students in Tibet who spent more than 2.5 h outdoors each day was higher in Tibet than in Chongqing (61.35 vs. 43.04%, respectively). Conversely, the percentage of students who spent more than 2.5 h on digital devices was 3 × higher among the students in Chongqing compared with those in Tibet (38.62 vs. 10.49%, respectively). Furthermore, greater monitoring of visual status among the Chongqing students was observed compared with the students of Tibet, with the optimal examination frequency being every 6 months.

Our observation showed that the myopia rate of children and adolescents living in plateau areas is lower than that of children and adolescents living in plain areas and is consistent with previously published results (24). There are multiple factors that may contribute to the observed differences in myopia rates. Accumulating evidence consistently demonstrates that time outdoors is a protective factor for myopia (25–27), with prolonged light exposure or increased light intensity promoting secretion of dopamine in the retina (28, 29). As a result, myopic progression is mitigated (30). Jin et al. (31) and He et al. (32) also revealed that an additional 20–40 min outside the classroom can help slow progression of myopia. For children with myopic parents, outdoor activity time with stronger sunlight intensity can protect these children from the onset of myopia (33). In Tibet, the average number of hours of sunshine and the light intensity are longer than in Chongqing. Moreover, we found that the percentage of Tibetan students who spent more than 2.5 h outdoors every day (61.35%) was higher than that in Chongqing (43.04%), which is consistent with the lower myopia rate observed among children and adolescents in our sample from Tibet.

Oculometric differences between different ethnic groups have been reported in a previous study (34). For example, Goh et al. (35) and Pan et al. (36) reported that Chinese populations have a higher prevalence of myopia than Indian and Malaysian populations in Singapore. Wang et al. (37) also found that myopia prevalence in the Han population (32.93%) is significantly higher than in the Tibetan population (21.64%) when they studied individuals over the age of 50 years living in Xining and surrounding areas. The same group also reported a higher age-adjusted prevalence of myopia in the Han population (31.8%) than in the Mongolian population (23.0%) in Inner Mongolia (38). Thus, ethnic differences appear to contribute to myopia incidence, and that was observed in the present study. However, most of the students included from Qamdo were Tibetan, so there were insufficient data to compare differences in myopia rates among different ethnic groups in the plateau regions. Previously, Wei et al. (39) reported that exposure to ambient air pollutants is associated with the pathogenesis of myopia. According to the official website of the Ministry of Ecology and Environment People's Republic of China (https://www.mee.gov.cn/), air pollution in Chongqing is consistently more serious than in Tibet. Thus, it is possible that environmental pollution may have further contributed to the difference in myopia rates observed in the present study.

Myopia appears to be strongly associated with near-work activities related to education and digital screen use (40). In the present study, students in Tibet had lower rates of digital device use, higher rates of choosing televisions and computers when using digital devices, and lower rates of using digital devices for more than 2.5 h compared with students in Chongqing. In a systematic review, a possible association between exposure to smart devices and an increased risk of myopia was observed (41). Ma et al. (42) also reported that time spent on digital screen devices was related to increases in myopia prevalence, whereas progression of myopia was slowed with use of projectors and televisions compared with use of mobile phones and tablets. Therefore, time spent using digital devices, and the type of digital devices used, contributed to the difference in myopia rates between plain and plateau areas. An association between myopia and years of education has also been reported in previous studies (43, 44). In China, children usually start primary school at an age of 6 or 7 years. However, the age of preschool education varies in different regions. In general, children in rural areas spend less time in preschool compared to urban areas, and less developed areas are characterized by shorter periods of preschool education than relatively developed areas. Similarly, there are also differences in educational pressure. Consistent with the results of previous studies (45, 46), we observed that the myopia rate of rural students in both plain and plateau areas was significantly higher than that of urban students. It is possible that earlier access to preschool education and greater educational pressure may have contributed to the higher rate of myopia among urban students than rural students, and it may represent an important reason for the higher rate of myopia observed in plain areas compared with plateau areas.

Interestingly, a recent study (47) showed that children ranging in age from 11 to 15 years exhibited a significantly enhanced risk of high myopia. These data indicate that younger children may represent a population that is more susceptible to myopia, and they suggest that myopic parents should pay greater attention to their children's eyesight in this age bracket. It has been observed that parents are generally nonchalant regarding health risks, incidence of myopia, and potential for a diagnosis of myopia (48, 49). Therefore, we propose that advocating frequent visual examinations may advance parents' awareness of myopia. In plateau areas, parents have been motivated to raise consciousness about myopic progression in children and the possibility of preventing myopia. However, this awareness and advocacy has not been matched with supporting measures (50). In the present study, the importance of parental awareness was demonstrated by investigating students' visual examination frequency. Other studies have revealed that parents worry about myopia in terms of time and financial burdens, in addition to concern regarding predisposition for high myopia (51). Economic and civilization standards are closely related to efforts to mitigate myopia progression. Parents also need to be directly engaged given their dominant role in determining the amount of indoor vs. outdoor activities that their children participate in Lee et al. (52). Therefore, parental behavior and impact with respect to children to mitiprogression is of particular concern and represents an acute area for awareness in societies.

### Study limitations

There are limitations associated with the present study. First, missing data and uncooperative participants were inevitable despite our efforts to perform a thorough survey of children and adolescents in the Chongqing and Tibet regions. As a result, broad 95% CIs were observed. Second, data regarding visual acuity prior to the COVID-19 pandemic were not obtained. Consequently, differences in the degree of myopia between the two regions with and without pandemic conditions could not be confirmed. Third, the other ethnic populations living in Tibet and Chongqing were not included in our present investigation. It is anticipated that these considerations can be addressed in future studies.

## Conclusion

The prevalence of myopia in Tibet was lower than that in Chongqing, accounting for gender, heredity, and regional factors. Outdoor activity time, selection of and time on digital devices, and parental awareness were factors associated with myopia progression. To identify and promote preventive strategies for myopia, risk factors for myopia and high myopia will continue to be examined. Moreover, this longitudinal epidemiological survey will continue to explore the prevalence of myopia in distinct altitudes.

## Data availability statement

The original contributions presented in the study are included in the article/supplementary material, further inquiries can be directed to the corresponding author.

## Ethics statement

The studies involving human participants were reviewed and approved by Ethics Board of the First Affiliated Hospital of Chongqing Medical University. Written informed consent to participate in this study was provided by the participants' legal guardian/next of kin.

## Author contributions

WWang and YX analyzed the data. LZ, BL, YJ, SZ, ZL, SY, LX, and TZ provided constructive advice for conception and data analysis. WWang wrote the manuscript. KH and WWan designed the study and reviewed the manuscript. KH, HH, FL, and LZ supervised the study. All authors contributed to the article and approved the submitted version.

## Funding

This work was supported by the National Natural Science Foundation of China (General Program, Grant Nos. 81100657, 81570832, 81870650, and 81970832), the Project Foundation of Chongqing Science and Technology Commission of China (General Program, Grant Nos. cstc2018jcyjA0429 and Cstc2021jcyj-msxmX0967), Chongqing Education Commission Project Fund of China (CQGJ17062B), and the Project of Chongqing Health Commission combined with Science and Technology Commission of China (2018GDRC008 and 2018MSXM003).

## Conflict of interest

The authors declare that the research was conducted in the absence of any commercial or financial relationships that could be construed as a potential conflict of interest.

## Publisher's note

All claims expressed in this article are solely those of the authors and do not necessarily represent those of their affiliated organizations, or those of the publisher, the editors and the reviewers. Any product that may be evaluated in this article, or claim that may be made by its manufacturer, is not guaranteed or endorsed by the publisher.
